# Supplementary material for: Structural and Biophysical Insights into SPINK1 Bound to Human Cationic Trypsin
Source: Int J Mol Sci. 2022 Mar 23;23(7):3468. doi: 10.3390/ijms23073468 (PMC8998336; doi:10.3390/ijms23073468)
Supplement: Supplementary file 1 [file ijms-23-03468-s001.zip › ijms-1637171-supplementary.pdf]

Supporting Information

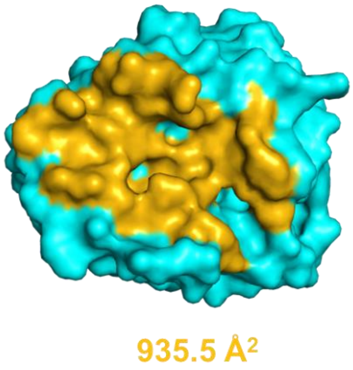

Figure S1. TRY1 p.S200A – SPINK1 WT interaction interface.

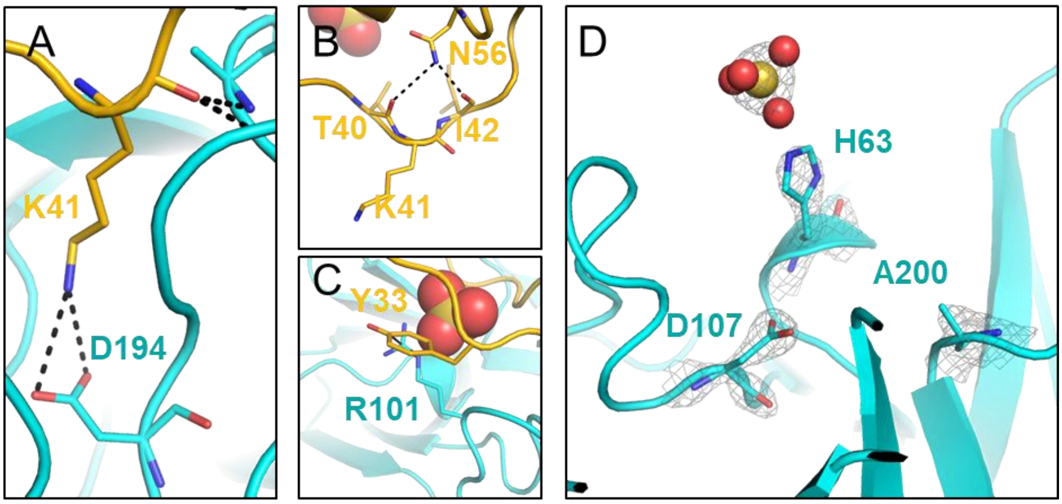

Figure S2. Interactions between TRY1 p.S200A and SPINK1 WT. SPINK1 WT is shown in yellow and TRY1 p.S200A in cyan. (**A**) Lys41 in SPINK1 interacts with the specificity determining Asp194 in TRY1. (**B**) Asn56 in SPINK1 stabilizes the binding loop by hydrogen bonding with Thr40 and Ile42. (**C**) Tyr33 in SPINK1 forms a cation – pi bond with Arg101 in TRY1 and is hence pulled outwards. (**D**) Catalytic triad of the TRY1 p.S200A – SPINK1 complex. In the complex structure His63 faces towards the sulfate ion and away from the catalytic triad. The 2Fo – Fc density map is shown 1.6 Å around the residues of the catalytic triad and is contoured at 1.0 σ.

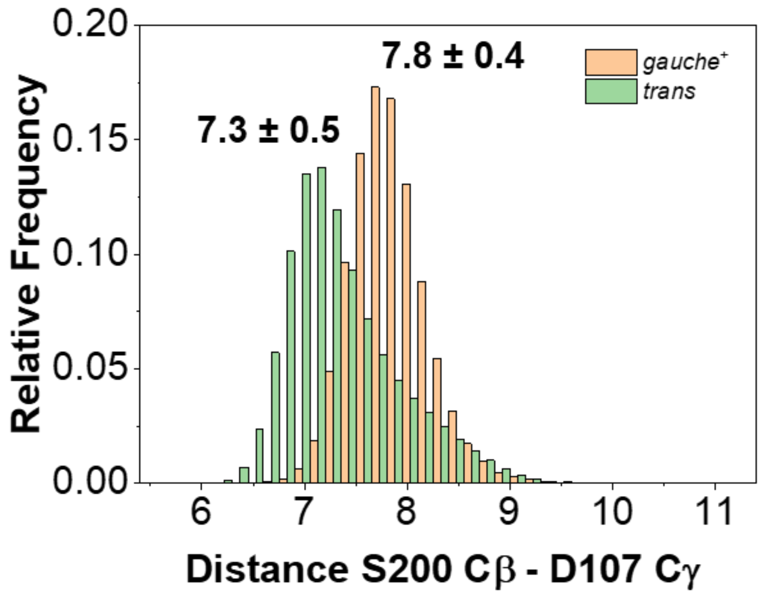

Figure S3. Distribution of distances between Ser200 and Asp107 of the catalytic triad during MDS. Distances were evaluated between complexes with His63 in *trans* (green) or *gauche*$^{+}$ (orange) conformation.

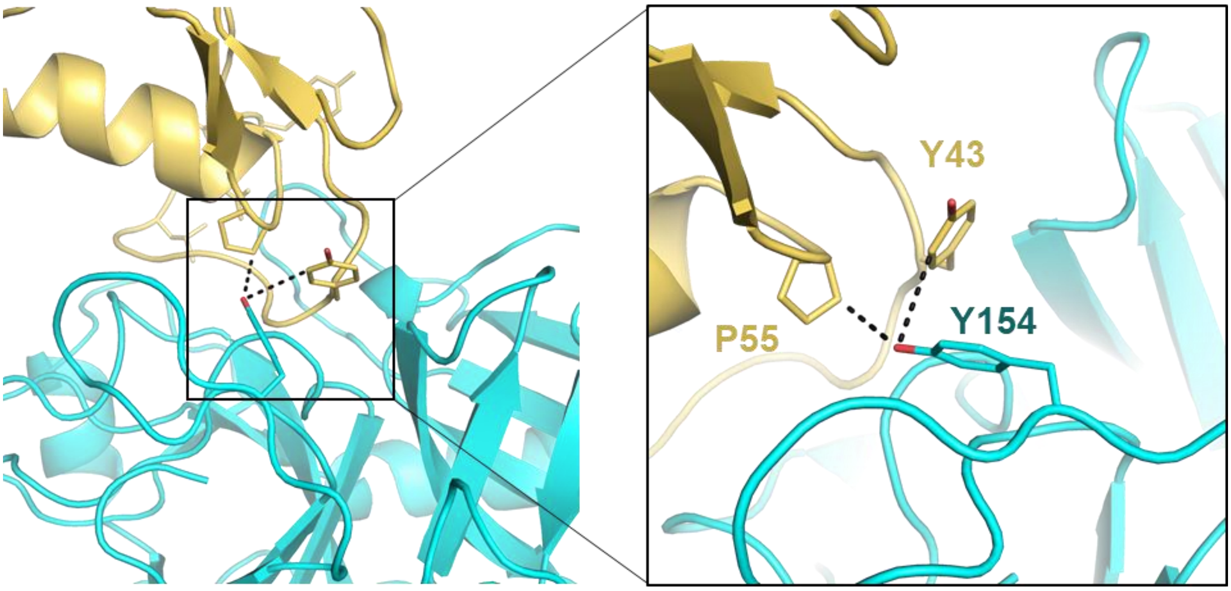

Figure S4. Interactions of Tyr154 with SPINK1 WT. SPINK1 WT is shown in yellow and TRY1 p.S200A in cyan. Tyr154 interacts with Tyr43 and Pro55 in SPINK1. Physiologically Tyr154 is sulfated, which weakens the interaction of SPINK1 with TRY1 due to steric hindrance.

List S1. Serine protease structures with p.S200A mutations. 4/74 structures (5.4 %) show alternative His63 conformations. Structures with alternative His63 conformations are highlighted in red.

4FXG	5JPM	2XW9	2XWB	5ZLZ	5BRR	4XSK	3PB1	2O8W	2O8U	4ZKS	5WXT	2PUX
2PV9	3K65	4HZH	4RN6	3SQE	3LU9	1TB6	4DT7	3SQH	3B9F	2B5T	1DM4	1JOU
1JMO	4H6S	4H6T	3KCG	4IS5	4ISL	5LYO	6BQM	6AOD	6TS4	6I44	1L4D	1BML
1DDJ	1L4Z	6D3Z	1MZA	1MZD	2GD4	5VOE	5VOF	2R9P	3L3T	5C67	3P92	4U32
6HAR	3L33	6GFI	5JBT	4U30	6BX8	3P95	4DG4	4B2B	4B2C	4B2A	4B1T	1OPH
4WXV	2RA3	3TGJ	1YLC	3FP8	1YKT	3FP7	1YLD	1K9O				
